# Creatinine clearance rate predicts all-cause and cardiovascular mortality in patients with MASLD: a Shanghai cohort study

**DOI:** 10.3389/fmed.2026.1740873

**Published:** 2026-03-25

**Authors:** Yingqi Hou, Yanqiu Huang, Chenghao Zhang, Wen Gu, Yadan Xu, Shuli Li, Jugang Wu, Jibin Liu, Honglin Liu

**Affiliations:** 1 Department of Clinical Laboratory, Zhongshan Second People’s Hospital, Zhongshan, China; 2 Institute of Oncology, Affiliated Tumor Hospital of Nantong University, Nantong, China; 3 Department of Laboratory Medicine, Affiliated Hospital of Changchun University of Chinese Medicine, Changchun, China; 4 School of Public Health, Shanghai Jiao Tong University School of Medicine, Shanghai, China; 5 The Second Department of General Surgery, Shanghai Ninth People’s Hospital, Shanghai JiaoTong University School of Medicine, Shanghai, China; 6 Department of Clinical Laboratory, The Third People’s Hospital of Liupanshui, Liupanshui, Guizhou, China

**Keywords:** cohort study, creatinine clearance rate, MASLD, mortality, Shanghai

## Abstract

**Background:**

Metabolic dysfunction-associated steatotic liver disease (MASLD) is a prevalent chronic liver condition globally, driving the need to identify prognostic risk factors. The creatinine clearance rate (CCR) serves as a sensitive early marker of glomerular filtration rate. Yet evidence regarding its specific association with mortality in MASLD patients remains limited.

**Method:**

The predictive value of the CCR for long-term survival was evaluated in a five-year cohort of adults with MASLD from Pudong District, Shanghai. Baseline characteristics were analyzed, and Kaplan–Meier survival analysis, LASSO regression, restricted cubic splines (RCS), and Cox proportional hazards models were employed to assess the relationship between the CCR and mortality risk, with sensitivity analyses conducted to test robustness.

**Results:**

Among 8,828 MASLD patients, a higher CCR was inversely associated with cardiovascular mortality (HR = 0.95; 95% CI: 0.95–0.96) and all-cause mortality (HR = 0.97; 95% CI: 0.96–0.97) (both *P* < 0.001). Compared to the lowest tertile (T1), the middle (T2) and highest (T3) tertiles showed lower risks (*P* for trend < 0.05). RCS analysis revealed a non-linear correlation for both outcomes (*P* for non-linearity < 0.05). Kaplan–Meier curves confirmed higher survival probabilities in the highest CCR tertile (log-rank *P* < 0.001). LASSO regression was used for variable selection and CCR was included in the multivariate prognostic model, suggesting that this index is valuable for model fitting and can serve as an auxiliary indicator for predicting mortality risk in patients with MASLD, which was supported by ROC analysis. Sensitivity analyses confirmed the stability of these findings.

**Conclusion:**

In this cohort of Shanghai adults with MASLD, a higher estimated creatinine clearance rate was independently associated with a significantly reduced risk of all-cause and cardiovascular mortality. CCR may serve as a valuable renal function-related prognostic marker for risk stratification in the MASLD population.

## 1 Introduction

The concept of metabolic associated fatty liver disease (MAFLD), formerly known as non-alcoholic fatty liver disease (NAFLD), was redefined in 2020. Metabolic dysfunction-associated steatotic liver disease (MASLD) is defined as a spectrum of liver conditions characterized by hepatic steatosis in the presence of at least one cardiometabolic risk factor. It represents the most common chronic liver disease worldwide (1, 2). Approximately 5%–20% of individuals with hepatic steatosis may progress to non-alcoholic steatohepatitis (NASH), among whom about 20%–30% of NASH patients may develop hepatic fibrosis or cirrhosis, and up to 2% of cirrhotic patients may eventually progress to hepatocellular carcinoma (3–5).

The creatinine clearance rate (CCR) refers to the kidney’s capacity to clear creatinine from the plasma per unit of time. Creatinine is a metabolic byproduct of muscle metabolism, primarily excreted via glomerular filtration. Therefore, CCR can reflect the filtration function of the kidneys and is an important clinical indicator commonly used to estimate the glomerular filtration rate (GFR) (6). Patients with MASLD may also experience renal impairment due to metabolic disturbances, which could lead to kidney injury and subsequently affect CCR. Moreover, the prevalence of renal insufficiency is notably higher among MASLD patients (7). In pediatric NAFLD/MASLD, renal impairment is commonly observed and is associated with disease severity. Additionally, hyperfiltration has been independently linked to significant liver fibrosis (8). Evidence suggests that nearly one-fifth of children show progression of renal insufficiency within two years, and an analysis based on National Health and Nutrition Examination Survey (NHANES) data indicated a significantly increased prevalence of renal insufficiency (RI) among adult NAFLD patients (9).

Renal dysfunction has a high incidence in MASLD. Thus, using the creatinine clearance rate, a common clinical measure of renal function, to develop a widely applicable mortality prediction model is highly valuable. This study screened MASLD patients from the Shanghai Pudong. We used baseline data to construct predictive models for all-cause and cardiovascular mortality. Our goal was to gain a more comprehensive insight into the role of CCR in MASLD development and patient prognosis.

## 2 Materials and methods

### 2.1 Study population

This study employed physical examination data from patients in Pudong District, Shanghai. This retrospective cohort study, based on the 2019 health examination cycle, initially included 62,812 participants screened for MASLD. We excluded 30,566 non-MASLD patients, and further excluded participants with missing age data (*n* = 3), missing weight data (*n* = 1,676), missing height data (*n* = 4), missing blood glucose data (*n* = 18,667), missing HDL-C data (*n* = 2,309), missing urea data (*n* = 712), and missing hypertension history data (*n* = 47), resulting in a final cohort of 8,828 patients (Supplementary Figure 1).

### 2.2 Definition of MASLD

The definition of fatty liver is based on the detection of hepatic steatosis by ultrasound. Metabolic dysfunction-associated steatotic liver disease (MASLD) was defined as having fatty liver disease without a history of excessive alcohol consumption (defined as ≥ 30 grams per day for men or ≥ 20 grams per day for women) and without other causes of hepatic steatosis or excessive alcohol consumption (≥ 30 g/d in men, ≥ 20 g/d in women) (10). The adult cardiometabolic criteria are defined as follows: (1) BMI ≥ 25 kg/m^2^ (23 kg/m^2^ in Asians) or WC > 94 cm in men, > 80 cm in women; (2) Fasting blood glucose (FBG) ≥ 5.6 mmol/L (100 mg/dL) or 2-h post-load glucose level ≥ 7.8 mmol/L (140 mg/dL) or glycated hemoglobin (HbA1c) ≥ 5.7% (39 mmol/L) or type 2 diabetes or treatment for type 2 diabetes; (3) Blood pressure ≥ 130/85 mmHg or specific antihypertensive drug treatment; (4) Plasma triglycerides (TG) ≥ 1.70 mmol/L (150 mg/dL) or lipid-lowering treatment; (5) Plasma high-density lipoprotein cholesterol (HDL-C) ≤ 1.0 mmol/L (40 mg/dL) in men, ≤ 1.3 mmol/L (50 mg/dL) in women or lipid-lowering treatment (10).

### 2.3 Use of creatinine clearance as an estimate of renal function

Creatinine is a breakdown product of dietary meat and phosphocreatine in skeletal muscle. Since the body’s production of creatinine depends on muscle mass, and creatinine is not eliminated extra-renally, under steady-state conditions, urinary excretion equals creatinine production regardless of serum creatinine concentration. Creatinine clearance approximates the glomerular filtration rate (GFR) when creatinine is freely filtered by the glomeruli. Therefore, the Cockcroft-Gault (C-G) formula is commonly used clinically to calculate creatinine clearance for estimating GFR and thus assessing renal function. The Cockcroft-Gault (C-G) formula uses the patient’s weight and gender to predict creatinine clearance rate (mL/min); for female patients, the result is multiplied by 0.85. The C-G formula primarily relies on age as the main predictor for creatinine clearance rate the expression of the formula is as follows: Creatinine clearance = [(140–Age) × Weight (kg) × (0.85 if women)]/[72 × Serum Creatinine (mg/dL)] (11).

### 2.4 Ascertainment of mortality

The follow-up duration for participants was calculated from study enrollment until either the occurrence of death or the cutoff date of 31 December 2024. Mortality information for the Pudong cohort was obtained from the Pudong CDC mortality surveillance system. All-cause mortality was defined as death from any cause, while cause-specific mortality was determined according to the International Classification of Diseases, Tenth Revision (ICD-10). Specifically, cardiovascular disease (CVD) mortality was defined based on ICD-10 codes (I00–I09, I11, I13, I20–I51, and I60–I69).

### 2.5 Assessment of covariates

Demographic and anthropometric indicators analyzed in this study—including sex, age, height, weight, body mass index (BMI)—as well as lifestyle information (smoking, alcohol consumption) and disease history (hypertension, diabetes), were all sourced from standardized physical examination questionnaires and physical examinations. Regarding laboratory indicators, biochemical data such as total cholesterol (TC), high-density lipoprotein cholesterol (HDL-C), low-density lipoprotein cholesterol (LDL-C), urea (UREA), glucose (GLU), and creatinine (CREA) were obtained from venous blood laboratory tests conducted during the same period. Age was included in the analysis as a grouped variable. Age groups were set as: ≤ 70 years, 71–80 year, 81–90 year, > 90 year referring to common clinical age stratification standards.

### 2.6 Statistical analysis

Categorical variables are expressed as percentages (%). CCR was divided into tertiles from lowest (T1) to highest (T3). Differences in categorical data among the three subgroups were assessed using the chi-square test. For normally distributed data, one-way analysis of variance (ANOVA) was performed. For data that were not normally distributed, the Kruskal–Wallis test was used. Restricted cubic splines (RCS) with three knots were used to illustrate potential non-linear relationships between CCR and all-cause mortality and cardiovascular mortality in MASLD patients. Cox regression analysis was employed to assess the relationship between CCR and all-cause and cardiovascular mortality in MASLD patients. Three models were developed with progressively increasing levels of adjustment for potential confounding factors: Model 1 was unadjusted; Model 2 adjusted for sex, age group,; Model 3 further adjusted for sex, age group, BMI, alcohol history, glucose, smoking history, uric acid, HDL-C, LDL-C, and TC. Cox proportional hazards models were constructed with creatinine clearance rate (CCR, estimated by the Cockcroft-Gault equation) and covariates. Time-dependent ROC curves and AUC values with 95% confidence intervals were calculated at 1-year, 3-year, and 5-year time points using the time ROC. The analysis was repeated using eGFR estimated by the CKD-EPI (11) equation to assess the impact of different renal function measures on predictive performance. Survival outcome probabilities were estimated using the Kaplan–Meier method and compared using the log-rank test. We also performed subgroup analyses to further clarify the impact of sex on clinical outcomes. Subsequently, we applied the least absolute shrinkage and selection operator (LASSO) to evaluate the importance of CCR in constructing a prognostic model. we calculated the integrated discrimination improvement (IDI) using the “survIDINRI” package and the continuous net reclassification improvement (NRI) using the nricens package in R, with a time horizon of 2,000 days. For IDI, perturbation resampling (500 iterations) was used to derive 95% confidence intervals. For continuous NRI, predicted event probabilities at 2,000 days were obtained from Cox proportional hazards models, and bootstrap resampling (500 iterations) was applied for 95% confidence interval estimation. The base model included age group, sex, BMI, total cholesterol, urea, smoking status, alcohol use, HDL-cholesterol, LDL-cholesterol, and fasting glucose. The extended model additionally incorporated CCR. Continuous NRI was used in preference to categorical NRI to avoid dependence on arbitrary risk thresholds. We estimated the 95% confidence interval (CI) of the AUC (12). To evaluate the potential impact of MASLD on the study outcomes, two sensitivity analyses were performed in this study. Firstly, we conducted a sensitivity analysis in the subgroup of patients without MASLD to further assess the robustness of the results. Secondly, all patients with MASLD were fully included in the analytical cohort, and multiple imputation was applied to handle missing data, followed by a repeated sensitivity analysis. The main analytical procedures were replicated via these two strategies to verify the stability of the core conclusions under different data processing conditions.

## 3 Results

### 3.1 Baseline characteristics of participants

**FIGURE 1:**
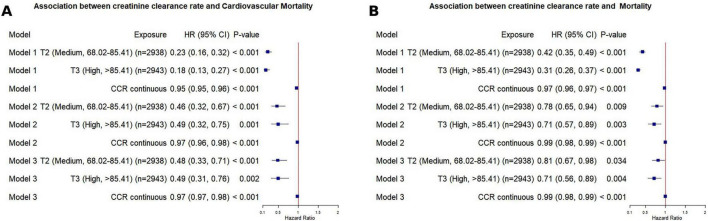
Association of CCR with cardiovascular and all-cause mortality in MASLD patients using Cox analysis. **(A)** CVD mortality; **(B)** All-cause mortality. Model 1 was unadjusted. Model 2 was adjusted for sex and age. Model 3 as further adjusted for sex, age, body mass index, smoking history, alcohol history, uric, glucose, high-density lipoprotein, low-density lipoprotein, total cholesterol. MASLD, metabolic dysfunction-associated fatty liver disease; T, tertile; HR, hazard ratio; CI, confidence interval.

This study included 8,828 participants, documenting 818 all-cause deaths and 248 cardiovascular deaths. The Table 1 presents core baseline characteristics after stratification by creatinine clearance rate (CCR) into low (T1), medium (T2), and high (T3) groups. The total sample size was 8,828, with 2,947 in the low group (T1), 2,938 in the medium group (T2), and 2,943 in the high group (T3). Significant differences were observed among the groups based on CCR in terms of age, sex, height, weight, smoking status, and mortality rates. Patients in the low CCR group (T1) were older, had a higher proportion of females, lower height and weight, and a higher all-cause mortality rate (15.7%), which was significantly greater than that in the high CCR group (T3), where participants were younger, had a higher proportion of males, and a lower mortality rate (5.2%). Additionally, urea levels were higher in the low group (T1) and relatively lower in the high group (T3), with all these differences being statistically significant (*P* < 0.0001) (Figure 1).

**TABLE 1:** Characteristics of the study population based on creatinine clearance rate (CCR).

Characteristics	Level	Overall	T1 (low)	T2 (medium)	T3 (high)	*P*-value
Number		8,828	2,947	2,938	2,943	
Age [mean (SD)]		71.95 (7.20)	77.25 (7.62)	70.67 (5.17)	67.94 (4.97)	< 0.0001
Gender (%)	Male	3,785 (42.9)	1,039 (35.3)	1,249 (42.5)	1,497 (50.9)	< 0.0001
	Female	5,043 (57.1)	1,908 (64.7)	1,689 (57.5)	1,446 (49.1)	
Height (cm) [mean (SD)]		161.61 (8.50)	158.13 (7.94)	161.85 (7.83)	164.85 (8.36)	< 0.0001
Weight (kg) [mean (SD)]		68.18 (10.38)	62.97 (9.04)	67.38 (8.81)	74.21 (9.97)	< 0.0001
Smoking (%)	No	7,308 (82.8)	2,663 (90.4)	2,405 (81.9)	2,240 (76.1)	< 0.0001
	Yes	1,520 (17.2)	284 (9.6)	533 (18.1)	703 (23.9)	
Alcohol (%)	No	7,636 (86.5)	2,723 (92.4)	2,537 (86.4)	2,376 (80.7)	< 0.0001
	Yes	1,192 (13.5)	224 (7.6)	401 (13.6)	567 (19.3)	
Diabetes mellitus (%)	No	6,525 (73.9)	2,157 (73.2)	2,249 (76.5)	2,119 (72.0)	0.0002
	Yes	2,303 (26.1)	790 (26.8)	689 (23.5)	824 (28.0)	
Hypertension (%)	No	8,752 (99.1)	2,915 (98.9)	2,915 (99.2)	2,922 (99.3)	0.2585
	Yes	76 (0.9)	32 (1.1)	23 (0.8)	21 (0.7)	
Urea [mean (SD)]		5.68 (1.57)	6.29 (1.94)	5.50 (1.25)	5.26 (1.22)	< 0.0001
TC [mean (SD)]		4.96 (1.02)	5.00 (1.08)	5.00 (1.01)	4.90 (0.96)	0.0001
HDL-C [mean (SD)]		1.29 (0.31)	1.30 (0.32)	1.29 (0.31)	1.28 (0.30)	0.3265
LDL-C [mean (SD)]		2.87 (0.77)	2.87 (0.83)	2.89 (0.76)	2.83 (0.69)	0.008
GLU [mean (SD)]		6.19 (1.84)	6.21 (1.81)	6.07 (1.73)	6.27 (1.96)	0.0001
CCR [mean (SD)]		77.32 (21.54)	54.84 (10.60)	76.56 (4.90)	100.59 (14.37)	< 0.0001
Death (%)	No	8,010 (90.7)	2,484 (84.3)	2,736 (93.1)	2,790 (94.8)	< 0.0001
	Yes	818 (9.3)	463 (15.7)	202 (6.9)	153 (5.2)	
Cardiovascular	No	8,580 (97.2)	2,774 (94.1)	2,897 (98.6)	2,909 (98.8)	< 0.0001
Disease death (%)	Yes	248 (2.8)	173 (5.9)	41 (1.4)	34 (1.2)	

CCR, creatinine clearance rate; GLU, glucose; TC, total cholesterol; HDL-C, high-density lipoprotein cholesterol; LDL-C, low-density lipoprotein cholesterol; GLU, glucose.

### 3.2 Associations between CCR and mortality risks by Cox regression analysis

**FIGURE 2:**
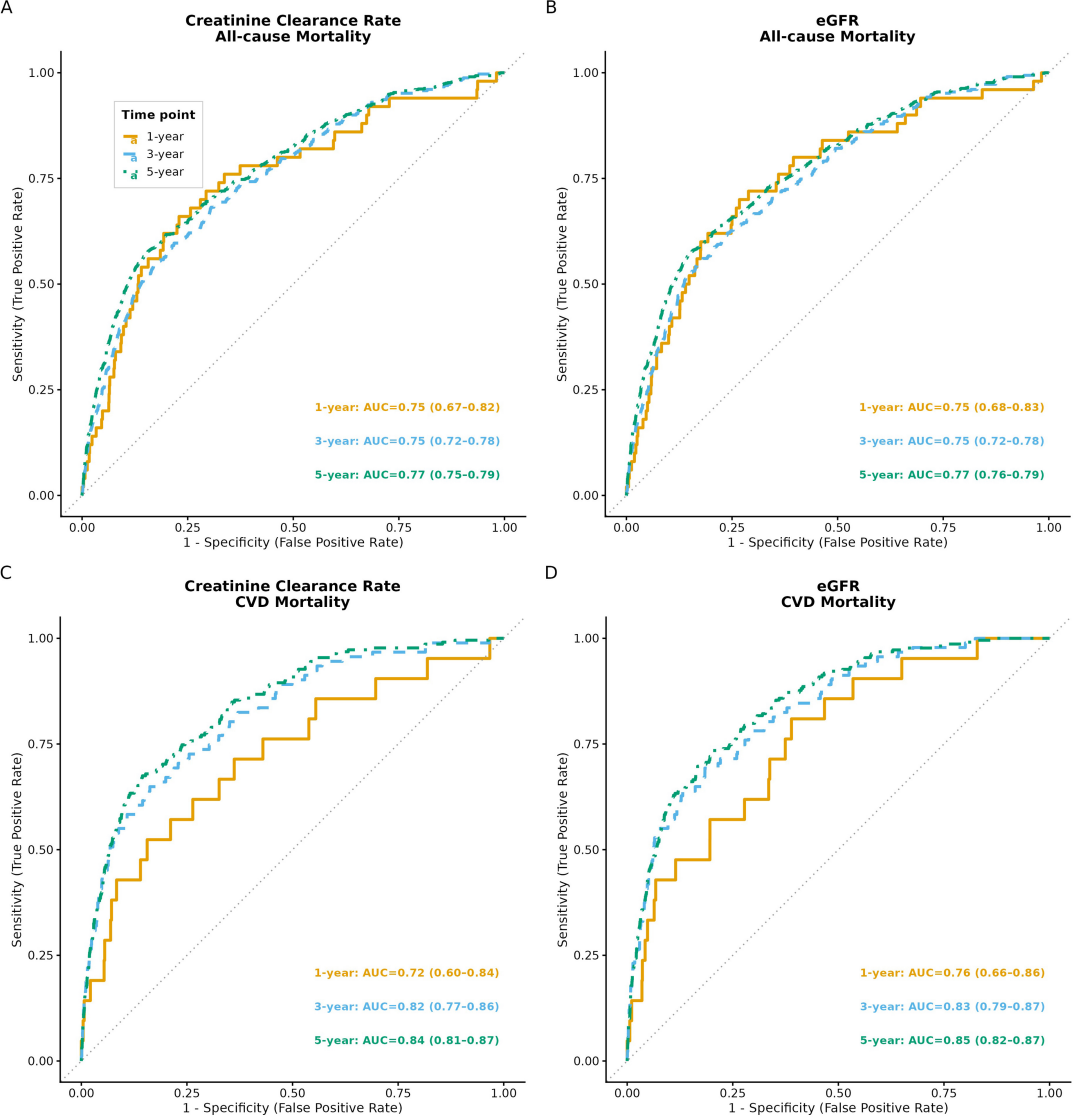
Time-dependent ROC curves for the predictive performance of Cox models using creatinine clearance rate and eGFR for all-cause mortality and CVD mortality. **(A)** Creatinine clearance rate for all-cause mortality; **(B)** estimated glomerular filtration rate for all-cause mortality; **(C)** CCR for CVD mortality; **(D)** eGFR for CVD mortality. AUC, area under the curve; CI, confidence interval.

In terms of cardiovascular disease (CVD) mortality, we observed a significant association between creatinine clearance rate (CCR) and CVD mortality. The hazard ratio (HR) was 0.95 (95% CI: 0.95–0.96, *P* < 0.001), indicating that each unit increase in CCR was associated with an approximately 5% reduction in CVD mortality risk. Based on CCR tertiles, cardiovascular mortality risk was significantly lower in the higher tertiles relative to the lowest one (*P* < 0.001). In the fully adjusted Model 3, compared to the lowest CCR tertile (T1), the medium (T2) and highest (T3) CCR tertiles were associated with reduced CVD mortality risk, with HRs of 0.48 (95% CI: 0.33–0.71) and 0.49 (95% CI: 0.31–0.76), respectively, both statistically significant (*P* < 0.001). For all-cause mortality, the HR was 0.97 (95% CI: 0.96–0.97, *P* < 0.001), indicating that each unit increase in CCR was associated with an approximately 3% reduction in overall mortality risk. Based on CCR tertiles, we observed a significant decrease in all-cause mortality risk with increasing CCR tertiles (*P* for trend < 0.001). In Model 3, compared to the lowest CCR tertile (T1), the medium (T2) and highest (T3) tertiles were associated with reduced all-cause mortality risk, with HRs of 0.81 (95% CI: 0.67–0.98) and 0.71 (95% CI: 0.56–0.89). Adding CCR to the base model improved risk discrimination for both outcomes. For all-cause mortality, the IDI was 0.0062 (95% CI: 0.0025–0.0111, *P* < 0.001) and the continuous NRI was 0.0281 (95% CI: 0.0065–0.0503, *P* = 0.012). For CVD mortality, the IDI was 0.0080 (95% CI: 0.0020–0.0190, *P* = 0.004) and the continuous NRI was 0.1785 (95% CI: 0.0776–0.2775, *P* < 0.001). The substantially larger NRI for CVD mortality compared with all-cause mortality suggests that CCR captures cardiovascular-specific prognostic information that is not reflected by conventional risk factors alone. Time-dependent ROC curve analysis based on the Cox proportional hazards model revealed that creatinine clearance (CCR) exhibited favorable predictive performance for all-cause mortality, with AUC values of 0.75 (95% CI: 0.67–0.82), 0.75 (95% CI: 0.72–0.78), and 0.77 (95% CI: 0.75–0.79) at 1-year, 3-year, and 5-year follow-ups, respectively. Its predictive efficacy for cardiovascular disease (CVD) mortality was even more prominent, with corresponding AUC values of 0.72 (95% CI: 0.60–0.84), 0.82 (95% CI: 0.77–0.86), and 0.84 (95% CI: 0.81–0.87), showing an upward trend with prolonged follow-up duration. To evaluate the impact of different renal function assessment approaches on the predictive performance of the model, this study simultaneously adopted the CKD-EPI 2021 equation to estimate the estimated glomerular filtration rate (eGFR) for constructing Cox models and plotting time-dependent ROC curves. The results indicated that the AUC values of eGFR and CCR for predicting all-cause mortality were highly similar at 1-, 3-, and 5-year follow-ups, and both indicators presented satisfactory predictive performance. For CVD mortality prediction, the AUC values of eGFR were slightly higher than those of CCR at all follow-up time points (Figure 2).

### 3.3 Restricted cubic spline (RCS) analysis

**FIGURE 3:**
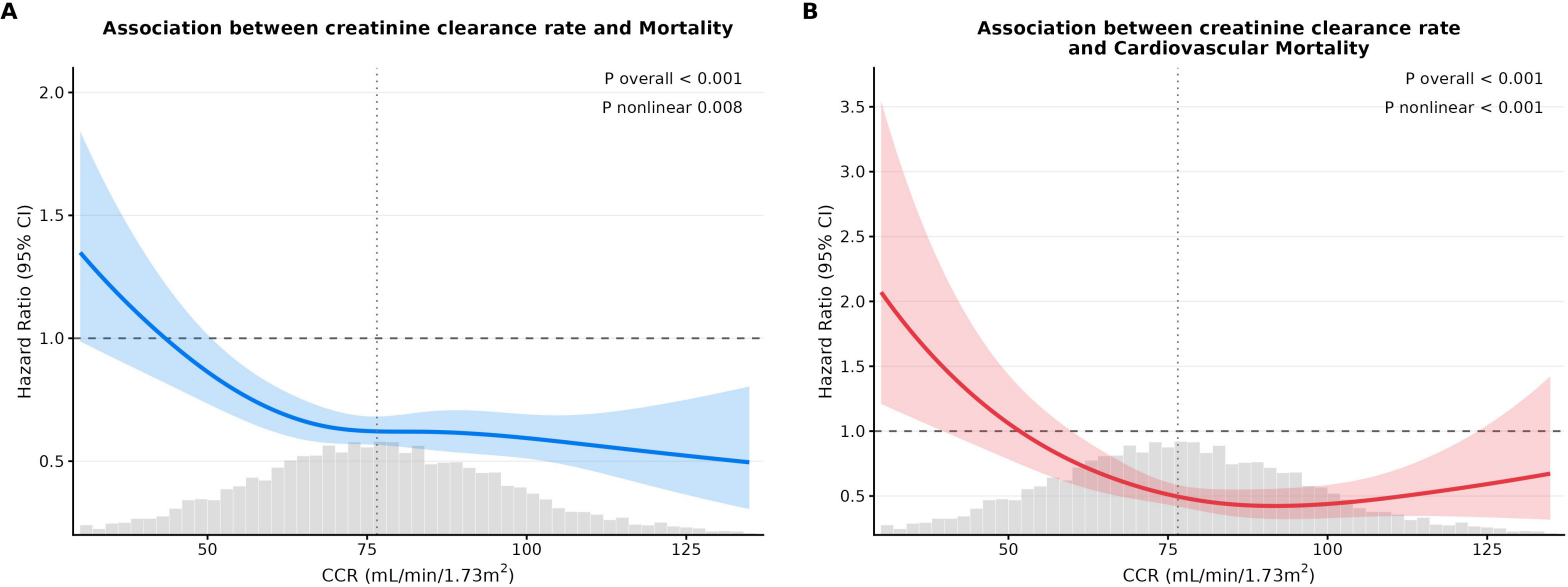
Association between creatinine clearance rate and all-cause and CVD mortality in MASLD patients visualized by restricted cubic splines. **(A)** All-cause mortality; **(B)** CVD mortality. Hazard ratios were adjusted for sex, age, body mass index, smoking history, alcohol history, uric, glucose, high-density lipoprotein, low-density lipoprotein, total cholesterol.

Restricted cubic spline analysis revealed that serum CCR levels indicated non-linear associations with both all-cause mortality (Figure 3A) and cardiovascular mortality (Figure 3B) in MASLD patients (all overall *P*-values < 0.001). Specifically, CCR showed an L-shaped relationship with all-cause mortality (*P* for non-linear = 0.008). For cardiovascular mortality (*P* for non-linear = 0.001), the curve displayed a distinct L-shaped characteristic, with the lowest risk observed at a CCR of approximately 75, a sharp increase in risk on the low-CCR side, and a relatively moderate increase on the high CCR side.

### 3.4 Kaplan–Meier survival curve analysis

**FIGURE 4:**
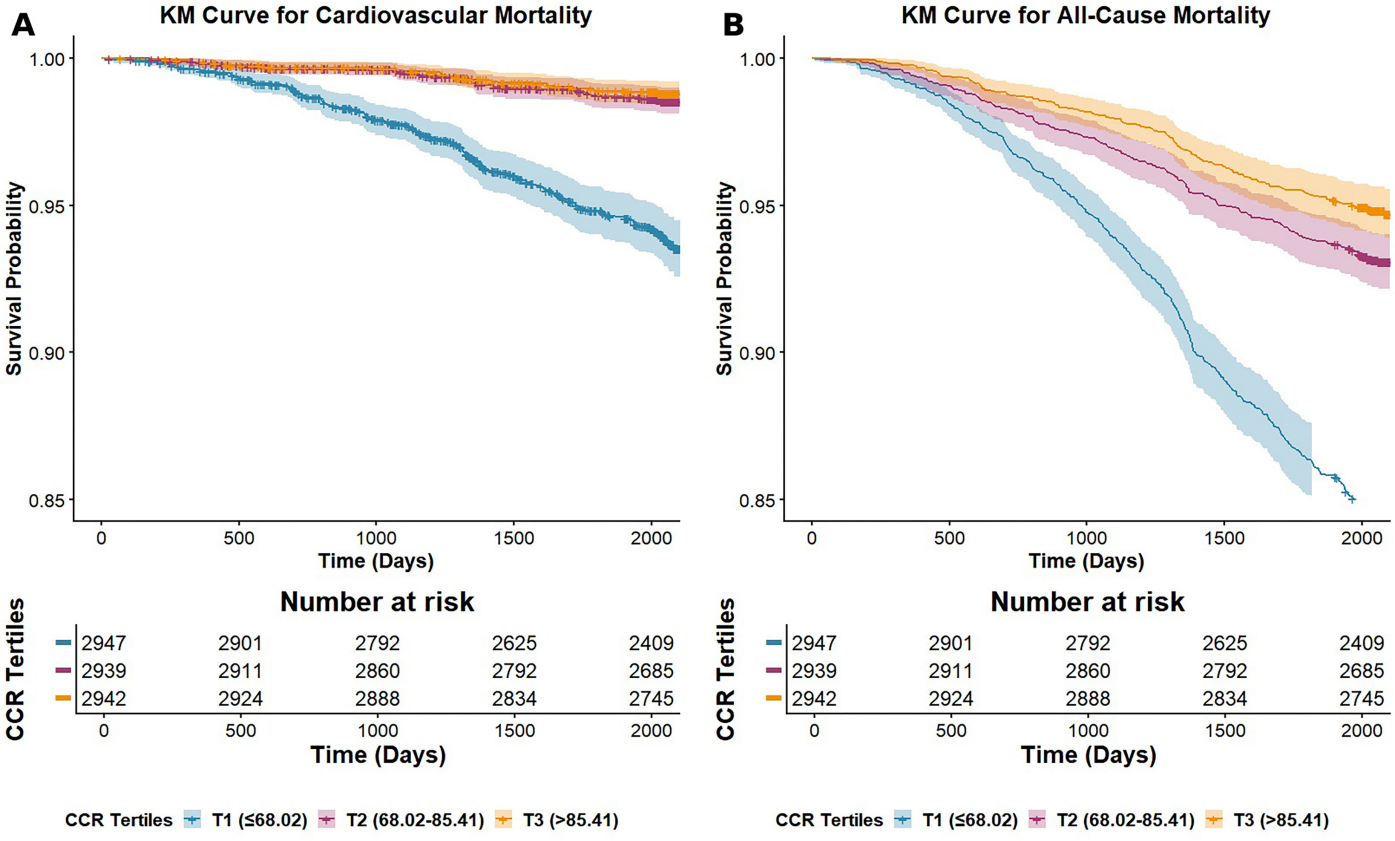
Kaplan–Meier curves of survival rates and the number of high-risk MASLD patients stratified by creatinine clearance rate tertiles. **(A)** CVD mortality; **(B)** All-cause mortality.

The Kaplan–Meier curves demonstrate a clear, graded inverse relationship between creatinine clearance rate (CCR) levels and mortality risk, with the T3 group (> 85.41) consistently exhibiting the highest survival probability for both CVD (A) and all-cause mortality (B) (Figure 4). A significant survival disadvantage is observed in the T1 group (≤ 68.02), as evidenced by its curve declining most rapidly and remaining lowest throughout the follow-up period in both panels. The early and sustained separation of the survival curves over 2,000 days suggests that CCR is a strong and persistent predictor of long-term mortality risk. The wider gap between the curves in Panel A (CVD mortality) compared to Panel B (all-cause mortality) implies that CCR has a particularly pronounced association with CVD mortality.

### 3.5 LASSO regression for predicting mortality in MASLD

**FIGURE 5:**
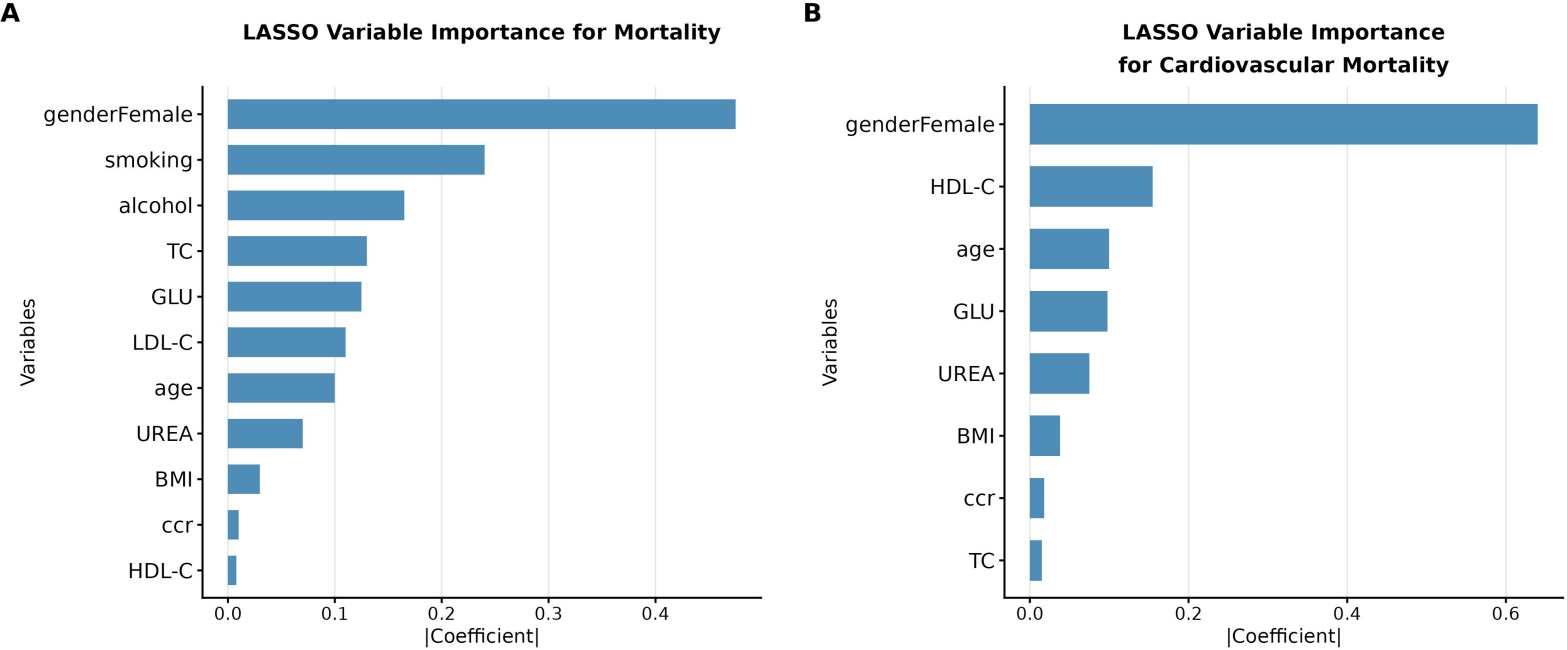
Contribution of creatinine clearance rate to all-cause and CVD mortality in MASLD patients based on LASSO regression. **(A)** All-cause mortality; **(B)** CVD mortality. LASSO, least absolute shrinkage and selection operator; CCR, creatinine clearance rate; GLU, glucose; TC, total cholesterol; BMI, body mass index; UREA, urea.

CCR was selected by the LASSO algorithm, albeit with a smaller coefficient than variables such as sex and glucose, suggesting that this indicator is associated with all-cause and cardiovascular mortality risk in patients with MASLD. This indicates that CCR can serve as a predictive factor in comprehensive risk assessment models (Figure 5). We employed the LASSO-Cox model for time-dependent prediction analysis of all-cause and CVD mortality.

### 3.6 Sensitivity analysis

**FIGURE 6:**
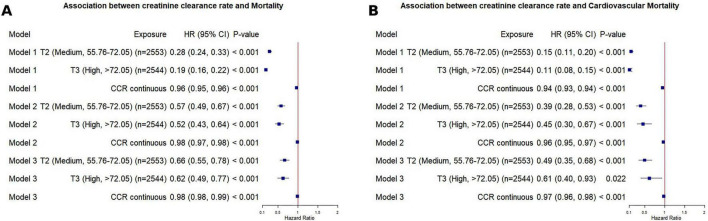
Sensitivity analysis of the association between creatinine clearance rate and all-cause and CVD mortality in the non-MASLD subgroup. **(A)** All-cause mortality; **(B)** CVD mortality.

**FIGURE 7:**
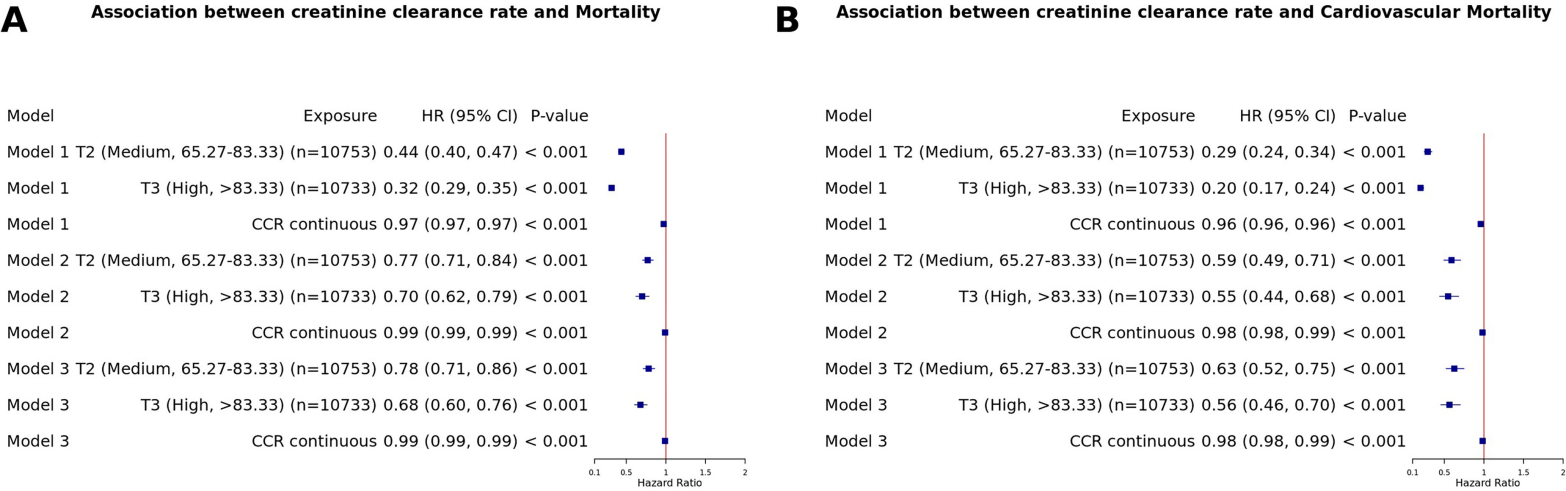
Sensitivity analysis of the association between creatinine clearance rate and all-cause and CVD mortality in MASLD patients after multiple imputation. **(A)** All-cause mortality; **(B)** CVD mortality.

To further validate the robustness of our findings, we conducted a series of sensitivity analyses. To verify the generalizability of the observed association, we first performed a sensitivity analysis among non-MASLD participants excluded from the main analysis; after excluding those with missing key variables, a significant inverse association between CCR and both all-cause and cardiovascular mortality endpoints was consistently detected in the remaining non-MASLD population (*P* < 0.001), which corroborated the reliability of the primary results (Figure 6). Furthermore, we conducted an additional sensitivity analysis by including all participants with MASLD participants without any data exclusion, and applied multiple imputation to address missing data before replicating the main analytical procedures. The inverse correlation between CCR and mortality outcomes remained statistically significant in this expanded cohort, further consolidating the stability and robustness of the core findings (Figure 7).

## 4 Discussion

This study conducted in Shanghai’s Pudong district represents the first systematic evaluation of the association between estimated CCR and long-term risks of all-cause and cardiovascular mortality in Chinese population with MASLD. The RCS analysis demonstrated a non-linear relationship between CCR and both all-cause and CVD mortality, indicating that the association followed a L-shaped pattern. The robustness of these findings was further confirmed through sensitivity analyses.

The significantly reduced risk observed in the high CCR group (T3) compared to the low group (T1) suggests considerable potential prognostic value of CCR in MASLD patients. The association between higher CCR levels and lower all-cause and cardiovascular mortality was consistently demonstrated through Kaplan–Meier analysis and Cox regression models adjusted for multiple confounding factors. This finding indicates that renal function status, as represented by CCR, may serve as an important supplementary indicator in the long-term prognostic assessment of MASLD patients. Compared to assessments focusing solely on liver-related metabolic parameters, CCR provides a more convenient reflection of systemic metabolic-excretory load and organ tolerance capacity, thereby offering additional value in predicting systemic risks, particularly cardiovascular risk. As an effective indicator of renal tubular function, CCR may hold significant importance in the long-term prognostic evaluation of MASLD patients. Compared to serum creatinine levels alone, CCR more accurately reflects glomerular filtration function, especially during early-stage renal impairment. Therefore, CCR may serve as a useful tool for assessing systemic metabolic burden and organ tolerance capacity in MASLD patients.

A Scottish study indicated that among MASLD patients, those with liver fibrosis face higher mortality risk, and when complicated by CKD, the mortality risk further increases (13). A UK Biobank-based study demonstrated that patients with both CKD and MASLD have higher risks of cardiovascular events and all-cause mortality compared to those without MASLD (14). Evidence also suggests that MASLD increases the probability of progression to end-stage renal disease in patients with pre-existing CKD (15, 16). Increased liver fibrosis has been shown to lead to higher all-cause mortality (17). Furthermore, our results indicate that high mortality and cardiovascular mortality rates in MASLD are associated not only with the presence of CKD but also with creatinine clearance rate. Therefore, estimating glomerular filtration function through creatinine clearance is crucial for addressing the increasing disease and mortality burden of MASLD. The rising prevalence of CKD, similar to MASLD, is expected to significantly impact global healthcare services in the medium term (18, 19). Once MASLD is diagnosed, active CKD screening should be implemented in these individuals. Early detection of CKD in MASLD patients enables additional interventions to modify disease progression, and robust multidisciplinary screening for MASLD patients with fibrosis can improve health outcomes.

Although the precise biological mechanisms linking CCR to MASLD mortality remain unclear, various potential mechanisms may be involved. We speculate that the association between CCR and mortality risk in MASLD patients may involve elevated creatinine levels indicating renal insufficiency, potentially reflecting systemic accumulation of metabolic toxins and electrolyte imbalances (20). Notably, the relationship between impaired renal function and MASLD is bidirectional. On one hand, chronic kidney disease (CKD) may exacerbate liver damage through the release of inflammatory factors and insulin resistance (21). On the other hand, MASLD-associated lipotoxicity and oxidative stress may directly impair glomerular filtration function (22). This bidirectional interaction is further supported by the significant interplay between the pathophysiological processes of the liver and kidneys, as well as their shared network of risk factors. Specifically, components of metabolic syndrome—including obesity, insulin resistance, type 2 diabetes mellitus, hypertension, and dyslipidemia—act as common upstream drivers that can simultaneously promote the development and progression of both MASLD and CKD (23). Additionally, the steatotic liver itself can produce uremic toxins similar to those associated with gut dysbiosis. This “double-hit” mechanism may explain the close association between reduced CCR (indicative of declined renal function) and elevated mortality in MASLD patients: when renal function is impaired, the clearance of these toxins is diminished, which further exacerbates systemic inflammation and organ damage, thereby forming a vicious cycle (23, 24). Furthermore, MASLD and CKD can collectively exacerbate insulin resistance, dyslipidemia, and hypertension through multiple pathways, including inflammation, oxidative stress, uremia, metabolic acidosis, physical inactivity, and further activation of the renin-angiotensin-aldosterone system (RAAS), thereby worsening metabolic syndrome (25–27). The underlying basis of these interactions may involve interactive feedback mechanisms between the liver and kidneys: impaired liver function can affect the kidneys through alterations in metabolism, blood flow, and inflammatory factors; conversely, declining renal function leads to waste retention and accumulation of oxidative mediators, which may increase metabolic stress on the liver. These processes collectively create an amplifying effect on organ damage (28), ultimately contributing to high patient mortality.

Given that mortality rates associated with both CKD and MASLD are on the rise (29), integrated co-management of these two highly prevalent conditions has emerged as an urgent unmet public health priority. Our study provides critical evidence that conventional screening strategies relying solely on binary assessment of CKD presence (i.e., positive/negative diagnosis) are insufficient to identify the full spectrum of high-risk patients. The results of this retrospective observational cohort study demonstrated that CCR was inversely associated with all-cause and cardiovascular mortality risk in patients with MASLD, suggesting that CCR may serve as an auxiliary reference indicator for prognostic risk stratification in these patients (30).

This subclinical decline in renal function often precedes overt kidney damage and warrants prompt, more proactive clinical intervention to prevent irreversible disease progression. The therapeutic landscape for comorbid liver and kidney disease is rapidly evolving. Emerging pharmacotherapies, including glucagon-like peptide-1 (GLP-1) receptor agonists and sodium-glucose cotransporter 2 (SGLT2) inhibitors, have demonstrated multifaceted benefits: not only do they reduce hepatic steatosis, but they also exert robust renoprotective effects through mechanisms such as improving renal hemodynamics, mitigating oxidative stress, and reducing tubulointerstitial injury (31, 32). To maximize the clinical value of these agents, future randomized controlled trials should incorporate pre-specified stratification of patients by baseline CCR levels and longitudinal CCR trajectories (33). This design will enable robust evaluation of the differential efficacy of these therapies across renal function subgroups, ultimately supporting personalized treatment strategies that reduce all-cause and disease-specific mortality in patients with concurrent MASLD and CKD. In this study, we adopted the Cockcroft-Gault (CG) formula to estimate creatinine clearance (CCR) for renal function assessment. As a clinically convenient and well-recognized tool, this approach demands no sophisticated calibration and is readily applicable in routine research and clinical settings. Of note, relevant evidence has indicated that for patients with metabolic abnormalities, CCR calculated by the CG formula represents a dependable marker for renal function evaluation.

This study has several limitations. First, as an observational cohort study, it cannot establish a causal relationship between CCR and mortality in MASLD patients, and the direction of causality remains unclear. Second, despite adjusting for multiple confounding factors (e.g., demographics, metabolic parameters), residual confounding from unmeasured variables (e.g., lifestyle habits) may exist. Measurement errors in key indicators like CCR and MASLD/CKD diagnosis may also introduce bias. Future interventional studies are needed to validate our findings and clarify the underlying causal mechanisms. Finally, this study has unavoidable selection bias, which is mainly attributable to missing relevant data among the enrolled participants. A substantial proportion of subjects were excluded from the final analysis due to incomplete baseline and laboratory indices, which was constrained by the availability of original retrospective data. This kind of sample attrition is common in real-world observational research.

## 5 Conclusion

In summary, higher CCR levels are associated with reduced all-cause and cardiovascular mortality. As a commonly used clinical indicator, CCR holds significant importance in the clinical management of MASLD patients. Through active monitoring of CCR levels, clinicians can more effectively identify high-risk mortality populations among MASLD patients, thereby optimizing monitoring strategies and guiding targeted interventions to ultimately improve patient prognosis.

## Data Availability

The raw data supporting the conclusions of this article will be made available by the authors, without undue reservation.

## Supplementary material

Supplementary Figure 1 Flowchart of the enrolled participants.
