# Supplementary figures and images for: Creatinine clearance rate predicts all-cause and cardiovascular mortality in patients with MASLD: a Shanghai cohort study
Source: Front Med (Lausanne). 2026 Mar 25;13:1740873. doi: 10.3389/fmed.2026.1740873 (PMC13057490; doi:10.3389/fmed.2026.1740873)

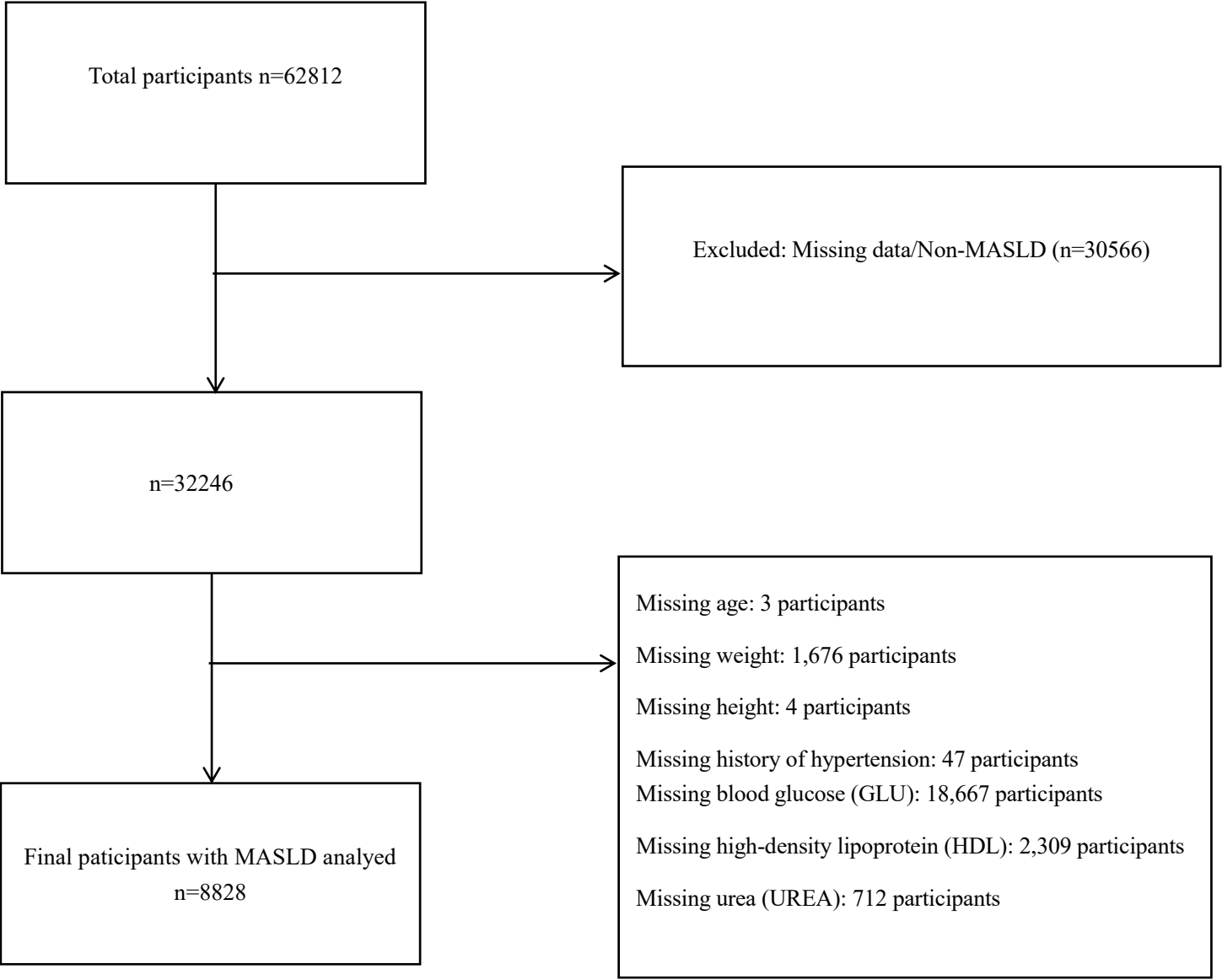

Supplement: Supplementary Figure 1 — Flowchart of the enrolled participants. [file Data_Sheet_1.pdf]
